# Pollen limitation and resource limitation affect the reproductive success of *Medicago sativa* L.

**DOI:** 10.1186/s12898-018-0184-x

**Published:** 2018-08-29

**Authors:** Min Chen, Xiao-An Zuo

**Affiliations:** Northwest Institute of Eco-Environment and Resources, CAS, Lanzhou, 730000 China

**Keywords:** Pollen limitation, Resource limitation, Pollinators, Seed set, *Medicago*

## Abstract

**Background:**

A large proportion of the flowers and ovules of plants do not develop into fruits and seeds. Plant reproduction may be limited because of pollen limitation and resource limitation. *Medicago sativa* L. is an ecologically important species in northwest China. We conducted a pollen supplementation experiment to determine the degree of pollen limitation in this species and detect the possible effects of resource allocation on pollen supplementation. We crossed two factors, pollen level (natural condition and hand pollinated) and resource level (control, water added, and fertilizer added), to estimate the effects of pollen addition and resource limitation on the opening of flowers and seed set. We also analyzed the floral characters, visitation frequency of pollinators and pollinator activity to estimate the effect of pollinators on the reproduction of *M. sativa*.

**Results:**

Our results indicated that addition of pollen to some flowers did not divert resources from other flowers and that the addition of pollen boosted the seed set per flower, with no effect on flower number. The primary effect of resource limitation was on the number of flowers produced; however, there was no significant effect on seed set per flower. These findings showed that pollen limitation was an important limiting factor for seed set. In addition, *Andrena lebedevi* Popov was identified as the most effective pollinator, and pollinator visiting and activity affected reproduction success in *M. sativa*.

**Conclusions:**

We found outcrossing was dominant in the breeding system and insect pollination played an important role in outcrossing. These findings have identified the dominant factor influencing seed set of *M. sativa*. This study aspires to contribute to a better understanding of pollen limitation, resource limitation and reproductive success.

## Background

Many plant species often produce more flowers and ovules than fruits and seeds [1, 2]. Several hypotheses have been presented to explain this phenomenon, including pollen and resource limitation [3, 4]. Pollen and resource limitation have received special attention because inadequate pollen and an insufficient availability of resources can reduce the reproductive success of plants [5]. An insufficient amount of pollen and compromised pollen quality have been demonstrated to result in reduced fruit and seed set, a phenomenon referred to as pollen limitation [6, 7]. Many studies have also indicated that plants are assumed to be limited by resources if the addition of resources increases fruit or seed set [8, 9]. In most flowering plants, pollen limitation and resource limitation are important constraints on reproduction [10, 11].

Pollination is the first stage in sexual reproduction, and pollination traits have an important influence on plant reproductive success [12]. Plant–pollinator interactions have been viewed as a key process in most flowering plant species [13]. In animal-pollinated plants, pollen delivery and the visiting frequency and activity of pollinators are major biotic factors influencing pollination success [14]. Pollinator abundance and activity decline as a result of a reduction in floral rewards if they then cannot meet the nutritional requirements of pollinators [15, 16]. The majority of pollen limitation occurs in cases when there are either not enough pollinators, or they are ineffective [7, 17].

A recent meta-analysis showed that estimates of pollen limitation are often biased when the flowers of a plant are manipulated, due to reallocated resources [18]. Many studies have indicated that pollen supplementation experiments may overlook the potentially confounding effects of reallocated resources on seed production [7, 19]. However, most studies only examine the consequences of pollen limitation on seed set [20], and few studies have measured the possible effects of resource allocation on the success of pollen supplementation [21, 22].

*Medicago sativa* has great potential as a forage plant and for medicinal use in arid regions [23]. An arid climate and harsh environment may affect plant–animal interactions because many self-incompatible species are highly dependent on pollinators in arid regions, and pollinators play an important role in reproductive success [24–26]. We measured pollen limitation and resource limitation in *M. sativa* in Linze. Our objective was to determine the relationship between floral traits and pollinators and how pollinator visitation and activity affect pollen limitation. Moreover, we identified the possible effects of resource allocation on pollen supplementation. We also estimated the relative impacts of pollen and resource limitation on the opening of flowers and seed set in *M. sativa*.

## Methods

### Study area and fragmentation experiment

This study was carried out from April 2015 to October 2017 in research patches in dry grasslands of the Linze Research Station in Gansu Province, China (37°50′–42°40′N, 100°02′–100°21′E). The studied patches are in Linze, and annual rainfall is approximately 130 mm.

**Fig. 1:**
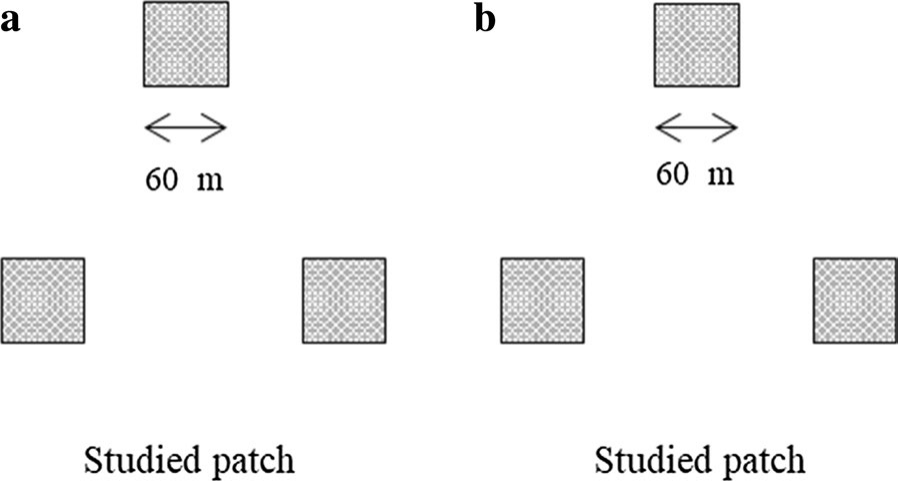
Experimental layout for two studied patches from 2015 to 2017. **a** One studied patch (three plots). **b** The other studied patch (three plots). Two studied patches, totaling six plots (60 × 60 m) were separated by mown vegetation (white area). The natural plants were symmetrically arranged and surrounded by undisturbed vegetation (gray area)

In this study, we focused on natural populations in an area where there were some typical plant species, such as *Reaumuria songarica* (Pall.) Maxim. and *Salsola passerina* Bunge. The flowering periods of *M. sativa* and these species of plants do not overlap. The original design consisted of two studied patches including a total of six plots in the dry grasslands. The three plots in each patch were symmetrically arranged and separated by mown vegetation (Fig. 1, white area). In addition, the average number of *M. sativa* plants was similar among the studied plots, and the distance between plots was approximately 100 m.

### Plant species

*Medicago sativa* is mainly distributed throughout western Gansu and Inner Mongolia provinces. This species is an important economic plant in northwest China. It has fascicled racemes, bisexual flowers in yellow or brown, four petals and ten stamens. In addition, the lengths of the corolla, bract and calyx in mm are (mean ± SD) 9.71 ± 0.62, 2.06 ± 0.19 and 3.83 ± 0.37, respectively.

### Floral characters and biology

To assess changes in the floral characters and floral biology, we labeled 50 flowers in the studied patches while they were still budding. For 2 weeks, we checked the flowers and noted the progression of the flowering stages. Video filming was conducted continually throughout anthesis of the labeled flowers, and data regarding the phases of flowering, time of anthesis, pollen viability, and pollen–ovule ratio were recorded.

Pollen viability was analyzed using pollen grains that were removed from the anthers of different individuals and stained with acetocarmine [27]. The stained pollen grains were counted using a stereo-zoom microscope. On each glass slide, we randomly selected five fields to observe and counted 100 pollen grains in total.

The pollen produced on the flower anthers was quantified using the hemocytometric method. To estimate the average number of ovules per flower, the ovules were carefully dissected out of the flowers and counted under the stereo-zoom microscope. The pollen–ovule ratio (P/O) was calculated according to Cruden’s [28] method:

$${\text{P}}/{\text{O }} = \frac{{\text{Pollen count /anther}} \times {\text{number of anthers / flower}}}{{\text{Number of ovules / flower}}}$$

### Pollen limitation

To study the degree of pollen limitation and whether the limited amount of pollen affected reproductive success, we conducted experimental hand pollination. For pollen supplementation, we harvested fresh pollen from other plants at least 10 m away from the experimental plants and then transferred it to the recipient stigma. The impact of pollen limitation on seed set was assessed using three treatment levels: pollen addition (PA), control (C), and non-manipulated (NM). Ashman et al. [5] found an effect of pollen limitation at the whole-plant level but did not evaluate the potentially mixed effects of resource reallocation. To detect the possible effects of resource allocation, we used two complementary controls, one using manipulated plants as a control and the other using non-manipulated plants as a non-manipulated treatment [21].

In total, there were 24 plants in the two patches. In each patch, we labeled 12 healthy plants at the same flowering stage and selected flower buds as experimental flowers. Moreover, one inflorescences was sampled from each targeted plant. On eight of the labeled plants, eight flowers on each plant were labeled from the central part of the plant, adding outcross pollen to the lower four flowers, which were classified as the PA treatment and leaving the upper four flowers alone as the C treatment. The PA treatment was carried out when the flowers were opened and the plants were hand pollinated. Four flowers on each plant of the four remaining targeted plants were also labelled from the central part of the inflorescence as the NM treatment. At the end of the seeding season, we used the seed set to estimate pollen limitation. Pollen limitation was estimated based on the seed set according to [29] as

$${\text{PL}}_{\text{C}} = { 1} - \left( {{\text{RS}}_{\text{C}} /{\text{RS}}_{\text{PA}} } \right)$$

where RS_C_ is the seed set under the control treatment, and RS_PA_ is the seed set under the pollen-added treatment. Positive values resulting from higher reproductive success in PA vs. C indicate pollen limitation, while zero or negative values indicate no pollen limitation [30].

### Pollen addition and resource limitation

To estimate the effects of pollen addition and resource limitation on the flower number and seed set, we marked 36 flowering plants, consisting of 18 plants under natural conditions and 18 hand-pollinated individuals, in six plots. The six plants in each plot were assigned to three treatments representing a factorial cross of two pollination levels (natural and hand pollinated) and three resource levels: (1) control, in which flowers experienced their natural resource environment; (2) water added, in which plants were given 60% more water (annual rainfall) than those in the control treatment before flowering; and (3) fertilizer added, in which a liquid nitrogen–phosphorus–potassium fertilizer (NPK, 9:2:6) was applied to the base of the plants once a month during the flowering season (1% v:v dilution, 20 ml per plant). These treatments were established to estimate the relative impacts of hand pollination and resource limitation on flower number and seed set. At the end of the seeding season, we collected the seeds and counted the number of opening flowers and seeds produced by the control, water added, and fertilizer added treatments in the laboratory [31].

### Pollinator visitation frequency and activity

During the flowering period, the times and frequencies of visits to *M. sativa* flowers were recorded over 2 weeks from 07:00 to 19:00. The pollinators collecting pollen and nectar were noted, and the behavior and type of pollinators were assessed using a DAT recorder. There were 120 h of observation for *M. sativa*. To determine the relationship between pollinator visitation frequency and the number of flowers opening, we marked a total of 18 flowering plants (9 plants in each patch) and conducted surveys of pollinators. Based on their visitation frequency and behavior on the flowers, insects were classified as effective pollinators or occasional pollinators. The pollinators were captured using insect nets, and the presence or absence of pollen grains adhering to their bodies was determined in the laboratory using a stereomicroscope. In addition, pollen preparations were made by rubbing a cube of fuchsin-stained jelly over the pollinator body [32]. The presence of pollen was regarded as proof of flower visitation. Species were identified by specialists. Additionally, three to six pollinators of each species were collected. The visitation frequency of pollinators (V_f_) was recorded and calculated according to the following equation:

$${\text{Visitation frequency}} = \frac{{{\text{Number of visits }}}}{{\text{Number of flowers}} \times {\rm observation time}}$$

### Breeding system

Field experiments were carried out in early April. A total of two hundred flowers were marked at the closed bud stage and were assigned to one of the five following five treatment groups in each plot: (1) natural pollination (control); (2) manual cross-pollination, in which the stigma of the emasculated flowers was hand-pollinated using pollen obtained from different flowers, and the flowers were bagged; (3) cross-pollination, in which the flowers were emasculated at the bud stage and open-pollinated; (4) self-pollination, in which the flower buds were covered with bags and kept under natural conditions until fruit maturation; and (5) emasculation and netting, in which the stamens were removed prior to the release of pollen and the flowers were covered in a fine mesh (1 mm^2^) to prevent insect visits.

### Data analyses

We used a general linear model to determine the effects of treatments (PA and C) and studied patches on seed set. The model used treatments and patches as fixed factors, and seed set as the dependent variables.

A general linear model was used to determine the relative impact of pollen added (natural and hand pollinated) and resource limitation (control, water, and fertilizer) on flower number and seed set. The model used pollen level and resource limitation treatments as fixed factors, opening flowers and seed set as the dependent variables.

A one-way ANOVA was used to compare visitation frequency and seed set between treatments. We also used regression to test whether the number of opening flowers affected the pollinator visitation frequency, the model used the pollinator visitation frequency as the independent variable and the number of opening flowers as the dependent variable. All analyses were performed using the statistical software package SPSS 19.0 for Windows.

## Results

### Floral characters and biology

In the studied area, the major blooming period of *M. sativa* occurred from May until July, and the flowering time of a single flower was 3 days. The period of peak flowering was from 09:30 to 14:00 h, and flowers were completely closed by 19:00 (Table 1).

**Table 1:** Floral characters of *M. sativa*

Floral characters	Observation
Flowering period	From May to July
Anthesis begins	07:30–08:00
Flowers completely open	08:30–10:00
Pollen release	08:00–14:00
Number of pollens/flower	3821.6 ± 327
Pollen ovule ratio (P/O)	296.3 ± 38.79
Flowers completely close	18:00–19:00

The pollen viability was as high as 57.6% or more on the first flowering day, and the pollen shedding time was short. After 2 days, the anthers and stigma were completely dry. *M. sativa* produced relatively large numbers of pollen grains and ovules, with a moderately high pollen–ovule ratio (Table 1). The pollen–ovule ratio is indicative of the degree of outcrossing and the mode of pollination. Therefore, the pollen–ovule ratio value of 296.3 ± 38.79 for *M. sativa* indicates that outcrossing was dominant in the breeding system.

### Pollen limitation

**Fig. 2:**
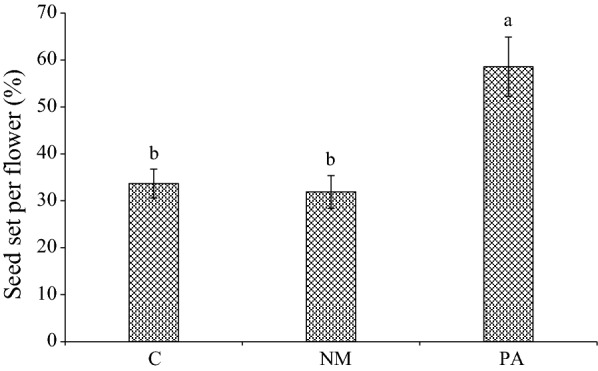
The mean seed set (mean ± SE) of *M. sativa* under pollen limitation treatments by one-way ANOVA with a Tukey-HSD test. Different letters show a significant difference at the 0.05-level. Vertical bars denote standard error. *C* control, *NM* non-manipulated, *PA* pollen addition

In the studied patches, the seed set did not differ significantly between the control and non-manipulated flowers, being 33.7 ± 3.8% in the control treatment and 31.9 ± 3.5% in the non-manipulated treatment. Pollen limitation also did not significantly differ between the control treatment (PL_C_ = 0.423 ± 0.036) and the non-manipulated treatment (PL_NM_ = 0.457 ± 0.039; P > 0.05). The seed set of flowers receiving pollen was 58.6 ± 6.1%, indicating that pollen supplementation significantly increased the seed set (P < 0.05; Fig. 2).

Moreover, our results show that pollen supplementation had a more significant effect on seed set than pollen limitation in the studied patches based on the comparison of seed set between the control and pollen-added treatments (P < 0.05; Table 2).

**Table 2:** Effect of treatments and studied patches on seed set of *M. sativa*

		Seed set			
		*df*	MS	F	P
PA vs. C	Treatment (T)	1	262.103	79.631	< 0.01
	Patch (P)	1	38.217	15.369	0.162
	T × P	1	7.316	1.276	0.087

*PA* pollen added, *C* control

### Pollen addition and resource limitation

**Fig. 3:**
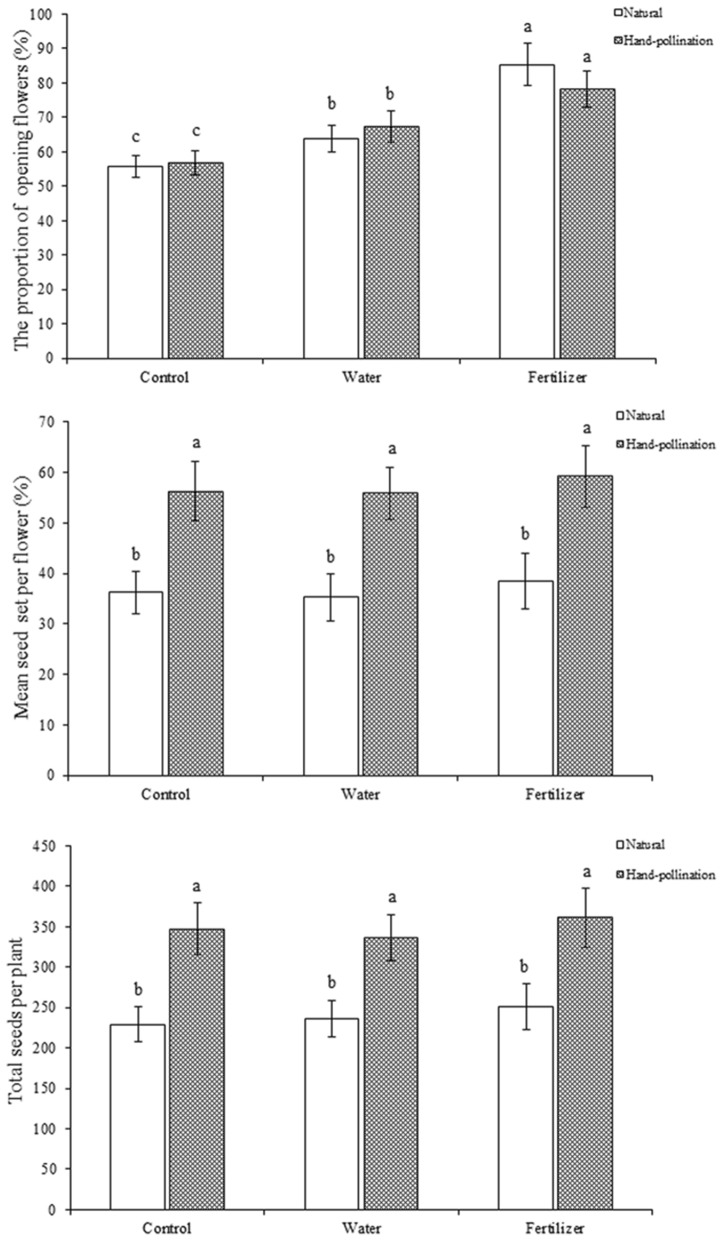
The proportion of opening flowers, the mean seed set per flower and total seeds per plant under resource limitation treatments of *M. sativa.* Different letters show a significant difference at the 0.05-level

Our results indicate that hand pollination increased the mean seed set per flower, which differed significantly between the control and fertilization treatments (P < 0.05). Under hand pollination, the proportion of open flowers in the control was similar to that in the fertilization treatments (P > 0.05; Fig. 3). We found that hand pollination could significantly affect the mean seed set per flower, with no effect on the proportion of open flowers.

Although both fertilization and hand pollination boosted the total number of seeds, they had different effects on the seed set per flower. Our outcomes showed that fertilization had no significant effect on the seed set per flower (P > 0.05; Fig. 3). We also found that the total number of seeds increased entirely due to fertilization (P < 0.05); watering alone had no detectable effect on the total seed production (P > 0.05; Fig. 3).

### Pollinator visitation frequency and activity

**Fig. 4:**
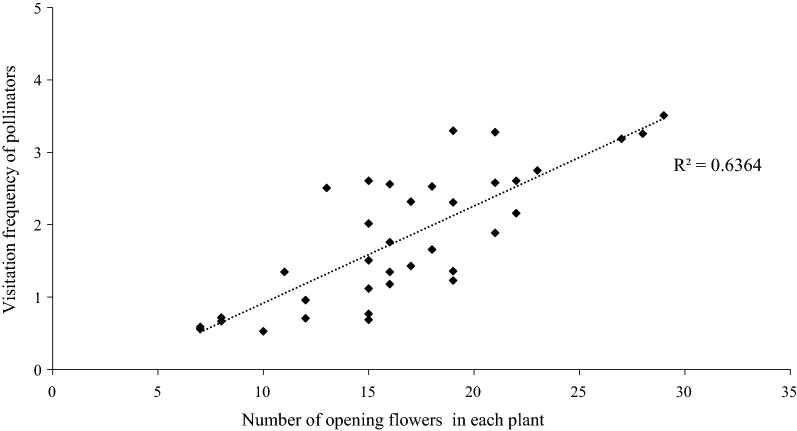
The relationship between visitation frequency and number of opening flowers. R^2^ index indicates the moderately positively correlated

In the studied patches, the flowers of *M. sativa* have a tripping mechanism, and pollinator activity acts as a tripping agent. The highest V_f_ of *Andrena lebedevi* Popov was 3.51 ± 1.6 (visits/hour). Moreover, our results showed a positive relationship between pollinator visitation frequency and number of open flowers, and pollinators tended to have more opening flowers (R^2^ = 0.6364; Fig. 4). This result may explain why *A. lebedevi* can easily carry and deposit more pollen than other pollinators from the anther of one flower to the stigma of another. Therefore, *A. lebedevi* was the most effective pollinator.

**Fig. 5:**
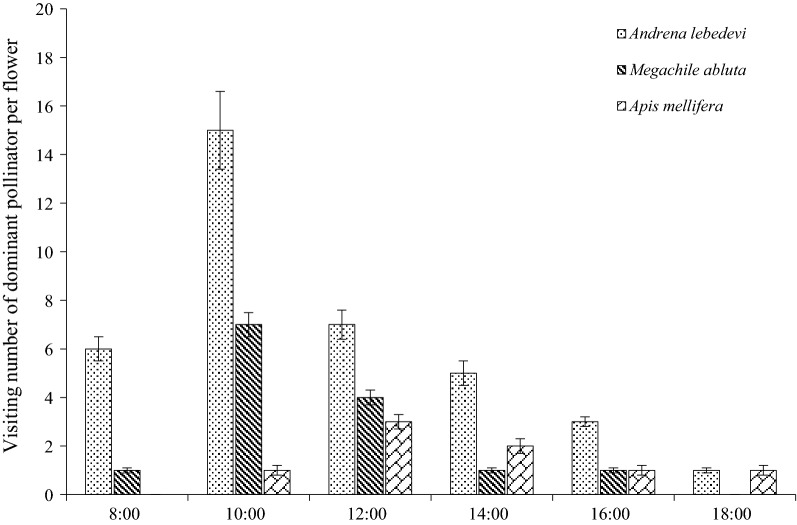
Frequency of pollinator visits to flowers of *M. sativa*

Our results indicate that bees comprised 86.9% of all 382 insects observed and that 63.6% of the pollinators were *A. lebedevi*, while 23.3% were *Megachile abluta* Cockerell. In addition, *Apis mellifera* Ligustica Spinola and *Pieris rapae* Linne were also recorded. In *M. sativa, P. rapae* (a butterfly) landed on the stamens of the flowers and only extracted nectar from the flower tubes using their proboscises. *A. lebedevi* collected pollen from the anthers, which accumulated on its legs and abdomen. Moreover, the visits of *A. lebedevi* peaked from 10:00 to 14:00 h, and this period coincided with the complete release of the pollen (Fig. 5). Therefore, *A. lebedevi* had a higher visitation number and a longer visitation time than the other pollinators (P < 0.05).

### Breeding system

**Fig. 6:**
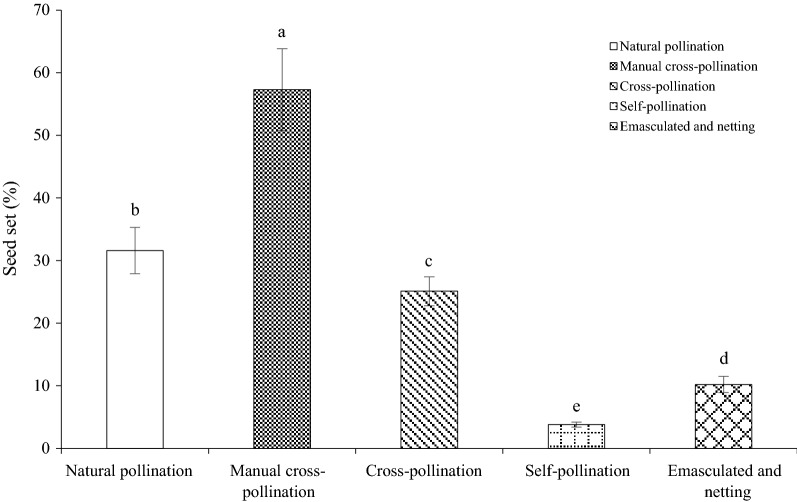
Seed set of *M. sativa* under different treatments

The seed set obtained in each pollination treatment is shown in Fig. 6. The seed sets were significantly higher in the manual cross-pollinated treatment than in the natural treatment (*df *= 1, P < 0.05), suggesting that outcrossing successfully promoted the pollination efficiency.

The seed set under emasculation and netting was only 10.2 ± 1.3%. In addition, the seed set in the cross-pollination treatment (emasculated and open-pollinated) was significantly higher than that in the emasculation and netting treatments (*df *= 1, P < 0.05). Our outcomes indicate that insect pollination plays an important role in the outcrossing system.

## Discussion

### Floral traits and pollinators

Floral traits and pollinator activity are largely considered to be co-adaptive attributes in which plants allocate resource to attract effective pollinators, and pollinators then evolve traits to better exploit floral resources [6]. In addition, floral traits maybe not only promote the more efficient transfer of pollen but also restrict other potential pollinators [33]. Our results indicate that there was a positive relationship between the pollinator visitation frequency and the number of open flowers. We also found that it is more efficient for pollinators to concentrate their visits to opening flowers because the filaments of plants dry easily in arid regions. In this study, flowers were completely open and pollen release occurred between 09:00 and 14:00 h, representing the important time for the pollination success of *M. sativa*. Moreover, this period coincided with the time of the highest activity of *A. lebedevi*. Therefore, *A. lebedevi* was identified as the most effective pollinator because this species can collect more pollen and visit more flowers than the other pollinators.

### Pollinator visitation and activity affect pollen limitation

Many studies have indicated that pollen limitation is strongly correlated with pollinator visitation [34, 35]. Pollinator visitation and activity have pervasive effects on pollination success or failure [6, 36]. A reduction in pollinators causes a decline in the amount of pollen delivered to the stigmas and reduces the probability of the transfer of cross pollen, resulting in reduced seed set [37]. In addition, low-quality pollinator activity can bring about pollen limitation by causing limited pollen availability and inefficient pollen transfer [38]. A similar pattern of pollen limitation has also been documented in *Ammopiptanthus mongolicus* (Maxim), reaffirming that pollinator activity affects pollen limitation [39].

Pollen limitation may be caused by quantity and quality limitation [40]. In animal-pollinated plants, insufficient pollen deposition is mostly caused by pollinator assemblage characteristics, such as pollinator visitation and abundance [6, 40]. Moreover, pollen quantity limitation is related to both pollinator frequency and pollination effectiveness [5]. We also found that insect pollination could increase efficiency of outcrossing and increase the seed set of *M. sativa*.

### Pollen limitation and reallocated resources

It has been shown that resource reallocation can inflate estimates of pollen limitation [7]. Plants may be able to reallocate resources among flowers, which leads to overestimation of the magnitude of pollen limitation by not accounting for the increased resources available to the treated flowers [2, 10]. Reallocation could occur primarily because flowers that obtain more pollen receive disproportionate resources, which has been the most commonly investigated condition, particularly in highly outcrossed plants [41]. Aizen and Harder [7] also found that resource reallocation may occur among fruits because of variation in the quantity or quality of pollen received.

Many studies have noted that if additional pollen is applied to only one flower or one inflorescence on a plant, resources may be reallocated away from untreated flowers for higher fruit set and seed production in the treated flower or inflorescence [5, 7, 10]. However, at the whole-plant level, supplemented and controlled plants may produce similar number of fruits and seeds. That is, the response to pollen supplementation by a polycarpic plant may come at the cost of future reproduction or survival, but this did not occur within the duration of the experiment [5]. We used flowers from non-manipulated plants to detect the possible effect of resource allocation on pollen supplementation [21]. Our results indicate that control flowers from non-manipulated plants had a lower seed set than control flowers from manipulated plants, suggesting that resource reallocation did not add pollen from other flowers.

### Effect of pollen limitation and resource limitation on plant reproduction

Plants are immobile and therefore typically rely on biotic vectors for the transportation of pollen for reproduction [5]. The majority of plant species rely on animal-mediated pollen flow to enhance pollen transfer among flowers [42]. Vázquez et al. [43] suggested that areas that are more frequented by pollinators could experience increased pollination rates and that visit frequency seems to be a good indicator of pollination success. In flowering plants, pollen limitation and resource limitation are widespread [10, 27]. Pollen supplementation may increase the seed set per flower in *M. sativa* because of this species’ need for the limited amount of pollen [6]. In *Apocynum venetum* L., pollen supplementation increased the seed set, and pollen limitation was the dominant limiting factor [41]. In this study, we also found that pollen addition could significantly boost the seed set per flower. We concluded that pollen limitation, rather than resource limitation, was responsible for the low seed set per flower.

Pollination is an essential process in the sexual reproduction of seeding plants, and pollination success is related to the identity of pollinators, as different flower visitors vary in their pollination effectiveness [44]. In our study, pollinators respond to variations in high resource availability, and their visitation and activity affected the reproductive success of *M. sativa*. Moreover, *M. sativa* was self-incompatible, and insect pollination played a more important role in the outcrossing system. These reasons may explain why high resource availability can increase the visitation frequency of pollinators and the efficiency of outcrossing in *M. sativa*.

## Conclusion

We have found pollen limitation was the dominant limiting factor for reproductive success. The primary effects of resource limitation was on the number of flowers produced, and there was no significant effect on seed set per flower. Our study also indicates that insect pollination plays an important role in outcrossing.
